# In Search of the Optimal Surgical Treatment for Velopharyngeal Dysfunction in 22q11.2 Deletion Syndrome: A Systematic Review

**DOI:** 10.1371/journal.pone.0034332

**Published:** 2012-03-28

**Authors:** Nicole E. Spruijt, Judith ReijmanHinze, Greet Hens, Vincent Vander Poorten, Aebele B. Mink van der Molen

**Affiliations:** 1 Department of Plastic Surgery, University Medical Center, Utrecht, The Netherlands; 2 Department of Otorhinolaryngology, Head and Neck Surgery, Free University Medical Center, Amsterdam, The Netherlands; 3 Department of Otorhinolaryngology, Head and Neck Surgery, University Hospital, Leuven, Belgium; Beijing Institiute of Otolaryngology, China

## Abstract

**Background:**

Patients with the 22q11.2 deletion syndrome (22qDS) and velopharyngeal dysfunction (VPD) tend to have residual VPD following surgery. This systematic review seeks to determine whether a particular surgical procedure results in superior speech outcome or less morbidity.

**Methodology/ Principal Findings:**

A combined computerized and hand-search yielded 70 studies, of which 27 were deemed relevant for this review, reporting on a total of 525 patients with 22qDS and VPD undergoing surgery for VPD. All studies were levels 2c or 4 evidence. The methodological quality of these studies was assessed using criteria based on the Cochrane Collaboration's tool for assessing risk of bias. Heterogeneous groups of patients were reported on in the studies. The surgical procedure was often tailored to findings on preoperative imaging. Overall, 50% of patients attained normal resonance, 48% attained normal nasal emissions scores, and 83% had understandable speech postoperatively. However, 5% became hyponasal, 1% had obstructive sleep apnea (OSA), and 17% required further surgery. There were no significant differences in speech outcome between patients who underwent a fat injection, Furlow or intravelar veloplasty, pharyngeal flap pharyngoplasty, Honig pharyngoplasty, or sphincter pharyngoplasty or Hynes procedures. There was a trend that a lower percentage of patients attained normal resonance after a fat injection or palatoplasty than after the more obstructive pharyngoplasties (11–18% versus 44–62%, p = 0.08). Only patients who underwent pharyngeal flaps or sphincter pharyngoplasties incurred OSA, yet this was not statistically significantly more often than after other procedures (p = 0.25). More patients who underwent a palatoplasty needed further surgery than those who underwent a pharyngoplasty (50% versus 7–13%, p = 0.03).

**Conclusions/ Significance:**

In the heterogeneous group of patients with 22qDS and VPD, a grade C recommendation can be made to minimize the morbidity of further surgery by choosing to perform a pharyngoplasty directly instead of only a palatoplasty.

## Introduction

The 22q11.2 deletion syndrome (22qDS) is the most frequent human microdeletion syndrome [1], with a frequency estimated around 1 in 4000 [2]. There is marked phenotypic heterogeneity among patients. The most common concerns during infancy include congenital heart disease, immune disorders, feeding problems, and hypocalcaemia. In toddlers and school age children, developmental delay and speech problems surface. In adolescents and adults psychiatric issues arise [3], [4], [5].

The speech problems are mostly attributed to velopharyngeal dysfunction (VPD). The velopharyngeal valve which normally separates the oral and nasal cavities shows incomplete closure resulting in feeding difficulties and hypernasal crying in infants and hypernasal speech in older children. Hypernasality can impair speech intelligibility with subsequent frustration and social withdrawal [6]. Language acquisition is often delayed [7], [8], [9], [10], [11], [12]. The etiology of VPD in patients with 22qDS includes palatal defects, adenoid hypoplasia, and platybasia which enlarge the pharyngeal gap [13]. Furthermore, nasendoscopic views of attempted velopharyngeal closure show pharyngeal hypotonia [14].

Patients with hypernasal speech which is resistant to speech therapy or patients with VPD based on anatomic deficits are candidates for velopharyngeal surgery. Surgeons aim to correct VPD by decreasing the size of the velopharyngeal gap by injecting fat in the posterior pharyngeal wall, lengthening the palate, mobilizing a pharyngeal flap (PF) that spans the center of the velopharyngeal gap but retains lateral ports, and/or rotating lateral flaps to reduce the velopharyngeal port diameter [15]. There is little evidence guiding the choice between these procedures.

Theoretically, PFs are only appropriate for patients with good lateral wall motion [16], [17], [18], [19], [20]. When there is good velar elevation and poor lateral wall motion, a sphincter pharyngoplasty (SP) or Hynes pharyngoplasty can be used, provided the level of the flap inset is high enough to provide velopharyngeal competence [21] and low enough to avoid hyponasality [22].

VPD treatment algorithms based on these theories state that surgical procedures should be tailored to preoperative findings such as velopharyngeal gap size and gap shape [15], [23], [24]. Patients with coronal closure patterns are predicted to benefit from SP [25] while patients with sagittal closure patterns are predicted to benefit from PFs [18], [23]. However, these recommendations are not based on clinical trials or systematic reviews.

Given both the costs and potential complications associated with surgery, it is important to help surgeons chose which patients to operate on and which procedure to employ [26]. Clinical trials comparing PFs to sphincter pharyngoplasties in nonsyndromic patients show no difference in outcome when treatment allocation is randomized [16], [27], [28] or tailored to lateral pharyngeal wall and velar motion [19], [29], [30]. Patients with 22qDS were excluded from these clinical trials, therefore the question whether creating a PF is more effective than an SP in resolving VPD remains unanswered for this population.

This study aims to determine whether in patients with 22qDS and VPD a particular surgical procedure results in a greater percentage of postoperative normal resonance by systematically reviewing the available literature. Sub-questions include which procedure results in less morbidity and whether tailoring the procedure to preoperative patient characteristics results in superior outcome. Ideally, these questions should be answered in a clinical trial. However, patient acquisition rates necessitate multi-center collaboration [31], and surgeon preferences for certain procedures limit participation [16]. This retrospective study contributes to further insight in the outcome of different surgical procedures.

## Methods

### Ethics

No ethical approval was required to conduct a systematic review of the literature. Approval was granted by the Institutional Review Board at the University Hospital in Leuven, Belgium to include unpublished data from a chart review.

### Searching

No protocol exists for this systematic review, nor was such a protocol prospectively registered in the Cochrane database. Studies were found via computerized searches of the MEDLINE and EMBASE databases and the Cochrane CENTRAL Register of Controlled Trials on 11-11-11. The search syntax included synonyms of 22qDS (Di-George OR DiGeorge OR “Di George” OR velocardiofacial OR 22q11* OR del22q11* OR “velo-cardio-facial” OR Shprintzen OR “catch 22”) and surgery for VPD ((fat AND inject*) OR palatoplast* OR Furlow pharyngoplast* OR velopharyngo* OR “pharyngeal flap” OR Honig OR Hynes). No limits were imposed on publication type, date, or language. Additionally, references of the relevant studies were hand-checked to confirm that no relevant publications were missed by the electronic search. Finally, data from personal unpublished files was included.

### Selection

The search hits were scanned for relevance using the inclusion criteria: (1) report outcome after surgery for VPD, and (2) report separate results for patients with 22qDS. Where relevance could not be determined based on title and abstract, the full-text was assessed.

### Validity assessment

The methodological quality of each study was appraised using criteria based on the Cochrane Collaboration's tool for assessing risk of bias in therapeutic studies [32]. One point was accredited for each positive criterion: (1) genetic confirmation of 22qDS, (2) inclusion of all patients with 22qDS and VPD who underwent surgery at the center, (3) the choice for the specific surgical procedure was randomized, (4) speech outcome was assessed at least one year postoperatively for all patients, (5) speech assessors were blinded to the surgical procedure, (6) the speech test was validated, and (7) results included the number of patients with postoperative normalized resonance. Patient inclusion criteria were collected to determine whether the study results could be generalized to all patients with 22qDS with VPD requiring surgery. Outcome assessment at least one year postoperatively was considered important since resonance takes at least a year to stabilize after surgery [21], [33], [34], [35], [36].

### Data abstraction

Data abstraction was completed independently. When patients with isolated VPD or other syndromes with VPD were included in studies, only data from patients with 22qDS and VPD were included in this review. Data was collected from the studies including patient age at surgery, prevalence of palatal anomalies, details of the preoperative imaging and whether this was used to tailor the procedure, specifics on the surgery, the length of postoperative follow-up time until speech was assessed, and speech outcome variables. The surgery was considered tailored when preoperative imaging studies affected the surgeon's choice for a particular surgical technique. For example, only patients with good pharyngeal lateral wall adduction received PFs, or the amount of pharyngeal lateral wall adduction determined the PF width.

Surgical procedures were categorized as either fat injection, Furlow, intravelar veloplasty (IVP), PF, Honig, SP, or Hynes. In a Honig procedure a velar retropositioning is combined with the creation of a PF. The pedicle of the flap tubes postoperatively, minimizing the obstruction [37]. A Hynes procedure is derived from a SP with high inset of the lateral flaps implying splitting of the palate [24].

Non-standardized reporting of speech scores impeded comparison of preoperative baseline characteristics and postoperative outcome, and different definitions were used to indicate ‘improved’ speech. Therefore, it was not possible to inventory the degree of preoperative VPD. Yet, where possible, the numbers of patients with postoperative normal perceptual resonance, nasal emission, and understandable speech were distilled from the studies. The definition of normal scores differed per study, introducing a bias that may affect the cumulative evidence.

### Quantitative data synthesis

To compare the outcome of the various procedures, the percentage of patients who attained normal perceptual resonance, normal nasal emissions, understandable speech, hyponasal speech, obstructive sleep apnea (OSA), and those requiring further surgery were included in a weighted ANOVA with weights based on the number of patients each outcome was measured in. Where there were significant differences, these were further tested using contrasts with a Bonferroni correction. The following pairs were compared: 1) fat injection versus Furlow or IVP since these less obstructive procedures tend to be performed on patients with some velopharyngeal movement, 2) fat injection versus SP or Hynes since both augment the posterior pharyngeal wall, 3) fat injection, Furlow, or IVP versus PF, Honig, SP, or Hynes since the previous tend to be less obstructive than the latter, 4) Furlow, or IVP versus PF, Honig, SP, or Hynes to compare palatoplasties to pharyngoplasties, 5) PF versus Honig to compare the effect of differing flap width to creating a narrow PF combined with palatal retropositioning, and 6) PF versus SP or Hynes, and 7) Honig versus SP or Hynes to compare the different types of pharyngoplasties. No assessment of publication bias was done.

## Results

### Search and selection

**Figure 1:**
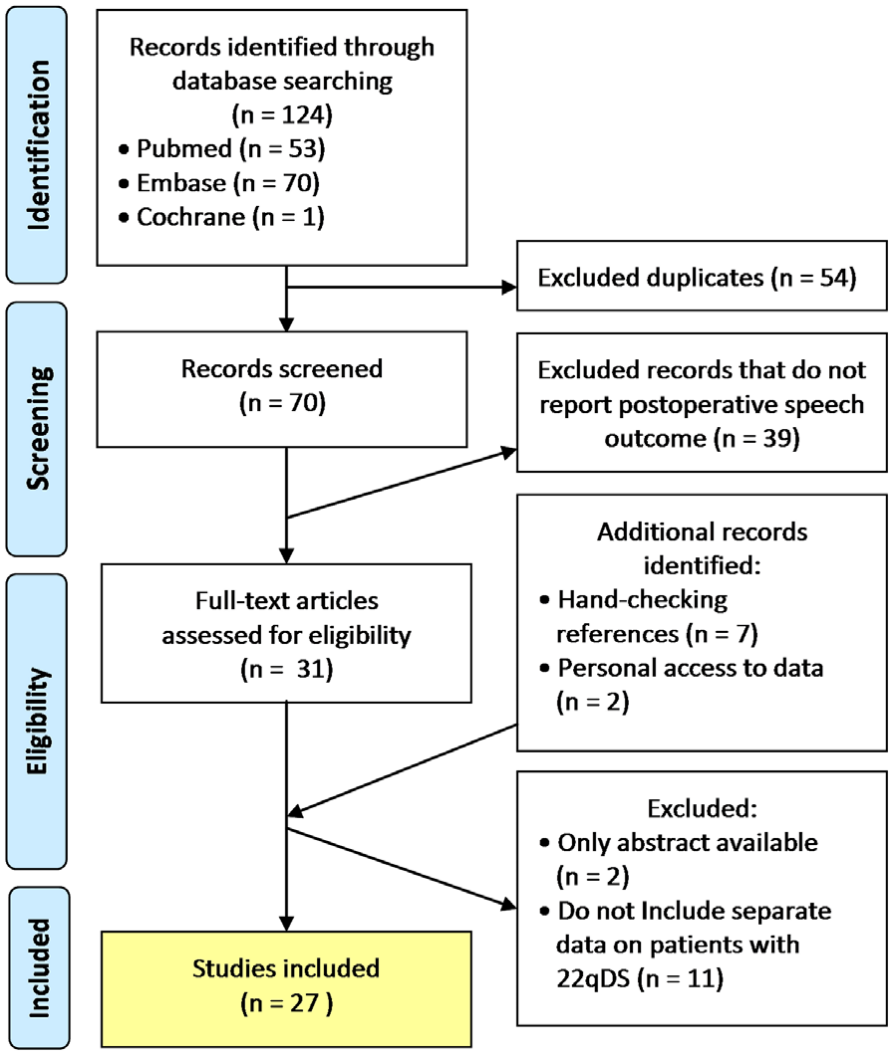
Study selection. Computerized search conducted on 11-11-11.

After filtering for duplicates, this electronic search strategy yielded 70 studies (Figure 1). Thirty-nine studies were excluded that did not report postoperative speech outcome. Hand-checking references yielded seven additional studies that report postoperative outcome but were missed by the electronic search because a synonym of 22qDS was not mentioned in the title or abstract, but only in the body text [38], [39], [40], [41] or a table [32], [33], [34]. For two of the relevant studies, only the abstracts have been published, hampering data extraction [42], [43]. Eleven studies were excluded that did not report separate results for patients with 22qDS. The authors have personal access to data from another relevant article which is still in press but has already been published in a dissertation [44] and to data from the University Hospital in Leuven, Belgium (Hens and Vander Poorten, co-authors). The combined electronic and hand-search strategy yielded 27 relevant studies for which data was accessible for analysis.

### Validity assessment

None of the studies met all the criteria indicating good methodological quality (Table S1). Genetic confirmation of the deletion was not always performed or reported. In most studies only a specific subgroup of patients who underwent surgery for VPD was reported. The choice for a particular surgical procedure was not randomized in any study. In only eight studies was the follow-up time at least one year for all patients. When loss to follow-up was reported, it ranged from 0–34%. In ten studies the outcomes of multiple surgical procedures were reported. In three of these studies the speech assessor was blinded to the surgical procedure [24], [45], [46].

In five studies postoperative speech was only reported in terms of improvement without data on the number of patients with normalization of resonance [33], [39], [46], [47], [48]. In three studies resonance was not one of the postoperative outcome measures [49], [50], [51]. These eight studies could not be used to answer the main question of this review, namely, whether a particular surgical technique leads to a higher percentage of patients with normal resonance postoperatively. However, these studies were still included in the analyses since they reported on the morbidity of the procedures.

In a handful of studies the inter- or intrarater reliability for the speech test were reported, indicating the validity of the test [21], [45], [50], [52], [53]. Others used the previously validated Cleft Audit Protocol for Speech [24] or Pittsburgh Weighted Speech Score [44], [48], [54]. No formal validation has been done for the Borel-Maisonny scale [33], [55], [56], the SISL (Screening Instrument for Cleft Palate Speech in Leuven) [Hens and Vander Poorten, unpublished data], or the test developed by the Dutch Association for Cleft and Craniofacial Anomalies [57].

Most study designs were outcomes research evaluating the speech of patients with 22qDS and VPD after undergoing surgery which is considered level 2c evidence [58]. Three studies were cohort studies in which patients with 22qDS and VPD who underwent surgery and those who did not were followed up. However, these studies were of poor quality deeming them level 4 evidence [58] due to ascertainment bias in recruiting patients to participate in the study [45], loss to follow-up >20% [6], or because follow-up times were not reported [59].

Data from all studies were used to determine which surgical procedure was most effective for resolving VPD in patients with 22qDS.

### Study characteristics

A comparison of the characteristics of the 27 studies, further subdivided by the groups of patients undergoing different procedures, revealed marked heterogeneity regarding which patients underwent surgery, the preoperative imaging, and the postoperative outcome measurements (Table 1, Table S2). Study sizes ranged from 1 to 44 patients. Many patients had palatal anomalies (57%, n = 282/494). Some had previous surgery on the palate or pharynx (n = 25) [35], [44], [53], [54], [55], [56], [57] or adenoid or tonsils removed (n = 69)[Hens and Vander Poorten, unpublished data] [6], [35], [44], [48], [53], [54], [57], [59], [60]. The patients included in the study by Argamaso et al [61] underwent surgery when they were on average twice as old as the patients in the other studies.

**Table 1:** Study characteristics and outcome, sorted by surgical procedure.

First author, publication year	Procedure	n	Palatal anomaly (%)	Age at surgery (mean years, range)	Follow-up (mean years, range)	Normal resonance (%)	Normal nasal emission (%)	Under-standable (%)	Hyponasal (%)	OSA (%)	Needing further surgery (%)
*Leuchter 2009 * [*55*]	fat injection	3	3/3 (100)	13.7 (11–17)	0.75 (0.4–1)	0/3 (0)	3/3 (100)	3/3 (100)	0/3 (0)	0/3 (0)	1/3 (33)
*Nicolas 2011 * [*56*]	fat injection	6	2/6 (33)	11.0 (7.7–17.9)	1.6 (1.0–2.6)	1/6 (17)	0/6 (0)	3/6 (50)	-	0/6 (0)	2/6 (33)
*Milczuk 2007 * [*46*]	Furlow	2	2/2 (100)	8.6 (6.4–10.8)	4.6 (3.2–6.1)	I	I	I	-	0/2 (0)	0/2 (0)
*d'Antonio 2001 * [*49*]	Furlow	4	4/4 (100)	-	-	-	-	-	-	-	4/4 (100)
*Rottgers 2011 * [*48*]	Furlow	13	13/13 (100)	(5.2)	2	I	I	-	-	0/13 (0)	2/13 (15)
*Perkins 2005 * [*39*]	Furlow	16	16/16 (100)	-	0.25	I	-	-	-	0/16 (0)	-
*Mehendale 2004 * [*24*]	IVP	14	14/14 (100)	5.7	(4 (0.6–9.17))	2/14 (14)	5/14 (36)	-	1/14 (7)	0/14 (0)	0/14 (0)
*Brandao 2011 * [*52*]	IVP	19	19/19 (100)	(9 (5–27))	1.4	4/19 (21)	-	-	-	-	-
*MacKenzie 1987 * [*59*]	PF	1	0/1 (0)	>3	-	0/1 (0)	0/1 (0)	-	0/1 (0)	0/1 (0)	1/1 (100)
*Argamaso 1994 * [*61*]	PF	2	-	25 (19, 31)	7 (5–9)	2/2 (100)	-	-	0/2 (0)	0/2 (0)	0/2 (0)
*Baylis 2008 * [*45*]	PF	3	2/3 (67)	<6.9 (<6.2–7.5)	-	0/3 (0)	-	1/3 (33)	1/3 (33)	-	-
*Rottgers 2011 * [*48*]	PF	4	4/4 (100)	(5.2)	0.8	I	I	-	-	0/4 (0)	0/4 (0)
*Witt 1998 * [*51*]	PF	5	5/5 (100)	6.4 (4–9)	-	-	-	-	-	-	4/5 (80)
*Brandao 2011 * [*52*]	PF	6	1/6 (17)	(9 (5–27))	1.4	3/6 (50)	-	-	-	-	-
*Arneja 2008 * [*21*]	PF	8	1/8 (13)	7.25 (4–11)	1	2/8 (25)	-	-	-	0/8 (0)	0/8 (0)
*Rouillon 2009 * [*33*]	PF	11	0/11 (0)	7.7	2	(10/11 (91)*)	-	-	-	0/11 (0)	0/11 (0)
*Goorhuis 2003 * [*60*]	PF	18	≥4/18 (22)	(4–6)	-	14/18 (78)	-	-	-	-	0/18 (0)
*Tatum 2002 * [*35*]	PF	20	19/20 (95)	6.2 (4–17)	0.5–2	18/20 (90)	18/20 (90)	-	0/20 (0)	0/20 (0)	0/20 (0)
*Ysunza 2009 * [*53*]	PF	20	20/20 (100)	(8 (5–25))	>0.5	12/20 (60)	-	-	0/20 (0)	0/20 (0)	-
*Swanson 2011 * [*54*]	PF	33	33/33 (100)	6.4 (4.4–19)	0.3 (0.1–8.8)	15/33 (45)	8/33 (24)	-	5/33 (15)	1/33 (3)	1/33 (3)
*Wang 2009 * [*50*]	PF	33	0/33 (0)	7.2 (4–17)	0.7 (0.2–2.1)	-	-	I	-	-	-
*Lipson 1991 * [*6*]	(likely PF)	32	19/32 (60)	<15.2)	>0.5	15/24 (63)	15/24 (63)	23/24 (96)	0/24 (0)	0/24 (0)	0/24 (0)
*Widdershoven in press * [*44*]	PF (33), SP (7)	40	13/40 (33)	7.5 (3.9–16.3)	2.4 (1–6.3)	28/40 (70)	18/40 (45)	-	0/40 (0)	1/40 (3)	4/40 (10)
*Rottgers 2011 * [*48*]	PF+Furlow	10	10/10 (100)	(5.2)	0.4	I	I	-	-	0/10 (0)	10/10 (100)
*Hens and Vander Poorten, unpublished data*	Honig	17	4/17 (24)	7.5 (3–23)	3.0 (1.0–7.8)	3/17 (17)	4/6 (67)	10/14 (71)	1/17 (6)	-	1/17 (6)
*Widdershoven 2008 * [*36*]	Honig	25	-	7.1 (3.8–13.6)	5	15/25 (60)	4/10 (40)	-	0/25 (0)	0/25 (0)	4/25 (16)
*Spruijt 2011 * [*57*]	Honig	44	23/40 (58)	6.0 (3.4–13.9)	7.0 (1.0–19.4)	23/44 (52)	14/27 (52)	34/36 (94)	-	0/44 (0)	8/44 (18)
*Baylis 2008 * [*45*]	SP	2	2/2 (100)	<6.1 (<4.8–7.4)	-	0/2 (0)	-	0/2 (0)	0/2 (0)	-	-
*Milczuk 2007 * [*46*]	SP	3	1/3 (33)	9.3 (5.7–12.5)	0.9 (0.3–1.3)	I	I	I	-	0/3 (0)	0/3 (0)
*Sie 1998 * [*25*]	SP	3	1/3 (33)	8.9 (5.2–12.7)	1.2 (0.6–1.8)	1/3 (33)	1/3 (33)	-	0/3 (0)	0/3 (0)	1/3 (33)
*Witt 1998 * [*51*]	SP	4	4/4 (100)	6.3 (5–8)	-	-	-	-	-	-	0/4 (0)
*Ysunza 2009 * [*53*]	SP	9	9/9 (100)	(8 (5–25))	>0.5	0/9 (0)	-	-	0/9 (0)	0/9 (0)	-
*Witt 1999 * [*62*]	SP	19	17/19 (89)	8.7 (4–16)	1	18/19 (95)	-	-	0/19 (0)	1/19 (5)	1/19 (5)
*Losken 2006 * [*47*]	SP	32	6/32 (18)	6.7 (1–15)	2.1	I	-	-	1/32 (3)	1/32 (3)	7/32 (22)
*Milczuk 2007 * [*46*]	SP+Furlow	9	0/9 (0)	9.5 (6.8–11.5)	1.4 (0.2–3.7)	I	I	I	-	0/9 (0)	0/9 (0)
*Hens and Vander Poorten, unpublished data*	Hynes	2	0/2 (0)	6.5 (5–8)	1.8 (1.2–2.3)	1/2 (50)	1/2 (50)	1/2 (50)	0/2 (0)	-	0/2 (0)
*Sie 1998 * [*25*]	Hynes	6	0/6 (0)	8.7 (4.7–13.4)	1.1 (0.2–2.9)	3/6 (50)	4/6 (67)	-	0/6 (0)	0/6 (0)	0/6 (0)
*Mehendale 2004 * [*24*]	Hynes	16	0/16 (0)	6.7	(4 (0.6–9.17))	3/16 (19)	6/16 (38)	-	2/16 (13)	0/16 (0)	3/16 (19)
*Mehendale 2004 * [*24*]	Hynes+IVP	11	11/11 (100)	6.7 (2.4–15.3)	(4 (0.6–9.17))	4/11 (36)	6/11 (55)	-	3/11 (27)	0/11 (0)	11/11 (100)

- : not reported; I: improvement; IVP: intravelar veloplasty; PF: pharyngeal flap; likely PF: pharyngoplasty not otherwise specified; SP: sphincter pharyngoplasty; ∧: include intermittent closure.

Pre-operative imaging included nasendoscopy and/or X-ray cephalograms or (video)fluoroscopy to confirm VPD or assess pharyngeal movement, including pharyngeal lateral wall motion, velar movement, gap size on attempted closure, and the closure pattern. Patients who underwent fat injections or palatoplasties tended to have better movement and smaller gap sizes than patients who underwent pharyngoplasties (Table S2).

The course of the carotid arteries was noted during nasendoscopy, using magnetic resonance imaging [21], [33], [35], [44], [54], [56], or intra-operatively. When an aberrant medialized course was found, some considered this a contraindication for surgery [33], other created a narrow PF [59], others suggested a palatoplasty would be safer than a pharyngoplasty [24], and others stated it had no consequence for the subsequent therapy [44].

At most centers the data accrued from imaging studies were used to tailor the surgery. Only patients who underwent a Honig velopharyngoplasty did not have a tailored surgery, Therefore, no subanalyses were performed comparing the outcomes of patients whose surgeries were tailored to those whose surgeries were not tailored.

In total, postoperative outcome was reported for 525 patients. Nearly half of the patients underwent a PF procedure. Lipson et al [6] did not specify what kind of pharyngoplasty was performed, but this was likely a PF since this was the most popular procedure in the early 1990s. Postoperative follow-up ranged from 0.2–19.4 years. Resonance was rated based on perceptual assessments by speech therapists using 2 to 20 point scales. Nasal emissions were assessed by auscultation or with mirrors and rated on 2 to 20 point scales. In some studies nasometry was used to assess the percentage of nasal resonance. Understandability was rated based on perceptual speech using 2 to 5 point scales or percentage scores. OSA was inventoried based on patient history, with subsequent polysomnography if necessary [44], [48], [54], [62]. In some studies speech outcome was reported following primary surgery for VPD and further surgery was recommended [49], [51], [59], [62], while in other studies speech outcome was reported following further surgery[Hens and Vander Poorten, unpublished data] [24], [25], [36], [44], [47], [48], [51], [54], [55], [56], [57].

### Quantitative data synthesis

**Figure 2:**
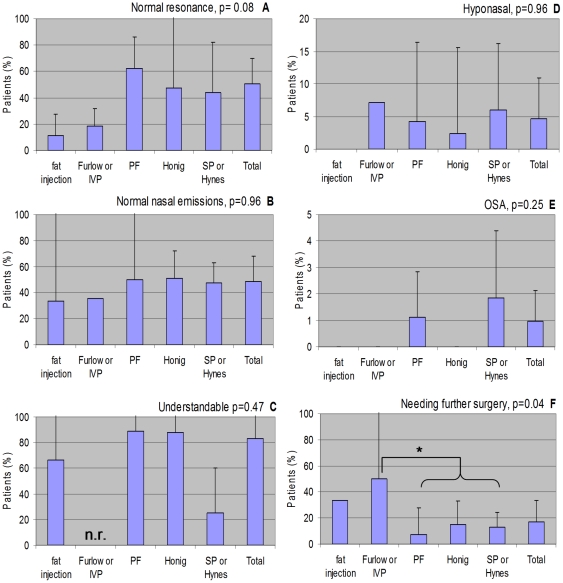
Outcomes by procedure. A) Normal resonance. B) Normal nasal emissions. C) Understandable. D) Hyponasal. E) OSA. F) Needing further surgery.

Overall, 50% of patients attained normal resonance, 48% attained normal nasal emissions scores, and 83% had understandable speech postoperatively. However, 5% became hyponasal, 1% had obstructive sleep apnea, and 17% required further surgery (Table 2, Figure 2). The standard deviations were large for many outcomes, and the variability between the standard deviations was large for the percentages with OSA and those needing further surgery (Levene's test p<0.05).

**Table 2:** Outcomes by procedure. Mean percentage of patients ± standard deviation (number of studies, number of patients).

	Fat Injection	Furlow or IVP	PF	Honig	SP or Hynes	All
*Normal resonance*	11±24 (2, 9)	18±19 (2, 33)	62±79 (11, 175)	48±100 (3, 86)	44±108 (8, 68)	50±98 (26, 371)
*Normal nasal emissions*	33±141 (2, 9)	36±0 (1, 14)	50±124 (5, 118)	51±37 (3, 43)	47±35 (5, 38)	48±79 (16, 222)
*Understandable*	67±71 (2, 9)	- (0, 0)	89±102 (2, 27)	88±73 (2, 50)	25±50 (2, 4)	83±77 (8, 90)
*Hyponasal*	0±0 (1, 3)	7±0 (1, 14)	4±35 (8, 143)	2±19 (2, 42)	6±30 (9, 100)	5±29 (21, 302)
*OSA*	0±0 (2, 9)	0±0 (4, 45)	1±6 (11, 181)	0±0 (2, 69)	2±8 (9, 108)	1±6 (28, 412)
*Needing further surgery*	33±0 (2, 9)	50±154 (6, 54)	7±66 (10, 142)	15±31 (3, 86)	13±34 (9, 94)	17±92 (30, 385)

IVP: intravelar veloplasty; PF: pharyngeal flap; SP: sphincter pharyngoplasty.

The diversity of quality, design, and patient populations of the included studies precluded a fixed or randomized meta-analysis. The heterogeneity could not be corrected for using a meta-regression since much data was missing, such as the amount of velopharyngeal movement. However, in an attempt to gain insight into overall trends in outcome, data was pooled according to the surgical procedures, grouping Furlow with IVP since both are palatoplasties, and SP with Hynes since the techniques differ only slightly. Widdershoven et al [44] report on 33 patients who underwent a PF and 7 patients who underwent an SP, but do not report the outcomes separately. The outcomes of all 40 patients were included in the PF group. For patients who underwent both a palatoplasty and a pharyngoplasty, most outcome measures were counted toward the pharyngoplasty groups. However, the need for further surgery was counted toward the palatoplasty group when this was part of the two-staged approach [24], [48].

Weighted ANOVA testing showed no significant differences with regard to speech outcome between the five procedure groups. There was a trend for the patients who attained normal resonance to differ between the groups (p = 0.08), with a lower percentage of patients attaining normal resonance after a fat injection or palatoplasty (11–18%) than after the more obstructive pharyngoplasties (44–62%).

Only patients who underwent PFs or SPs incurred OSA, yet this was not statistically significantly more often than after other procedures (p = 0.25).

The need for further surgery differed significantly between the five procedure groups (p = 0.04). Further testing with the contrasts and Bonferroni correction revealed that the difference was only significant between the patients who underwent a palatoplasty and those who underwent a pharyngoplasty (50% versus 7–13%, p = 0.03).

## Discussion

By systematically reviewing the available literature, data were presented and analyzed from 27 studies including 525 patients with 22qDS and VPD who underwent surgical correction. All surgeries except the Honig were tailored based on preoperative imaging. Overall, 50% of patients attained normal resonance. Fewer patients who underwent only a palatoplasty tended to attain normal resonance and more needed had a greater need for further surgery compared to than patients who underwent a pharyngoplasty. Therefore, the evidence suggests that for patients with 22qDS and VPD the morbidity of further surgery can be minimized when the cleft team decides a pharyngoplasty should be performed directly instead of only a palatoplasty. This is also the feeling the senior surgeon authors (ABM, VVP, GH) of this manuscript hold. VVP almost always chooses an extensive modified Honig procedure with supraperiosteal retropositioning and a cranially-based large PF in patients with 22qDS.

### Limitations

As aforementioned, questions about treatment efficacy should ideally be answered in a clinical trial. In a multi-center randomized controlled trial with nonsyndromic patients, 146 patients per procedure group were calculated to be required to find a 20% difference in outcomes between patients who underwent a PF and those who underwent a SP. However, the trial was terminated prematurely due to lower referral rates than predicted, changes in preoperative assessment leading to referrals for more nonsurgical interventions, and surgeons' growing preference for palate re-repair [16]. Among patients with 22qDS, larger variance is expected, necessitating even more patients per procedure group.

Given logistic hurdles, a practical solution to gain insight into trends requires turning to lower level evidence which is confounded by bias. For example, the 22q11.2 deletion was not genetically confirmed in all studies, most studies only included a specific subgroup of patients with 22qDS who required surgery to treat their VPD, and speech was only tested blindly and using a validated test in two studies [24], [45] (Table S1). The outcome of some pharyngoplasties may have been wrongly attributed to those pharyngoplasties since some patients underwent palatoplasties or multiple pharyngoplasties, either prior to being referred for the reported procedure [35] or as further surgery [51], [57]. Unfortunately, there was no data on the duration and intensity of postoperative speech therapy. Finally, when data are pooled there is a chance that the conclusions are misleading [63]. Therefore, raw data from each study are presented to allow readers to draw their own conclusions.

### Patients

When considering the management of VPD in patients with 22qDS, as for all patients with VPD, there are both conservative and surgical options. No randomized studies have been conducted to compare the effect of the natural history of speech development to the effect of intervention since leaving VPD untreated is considered ethically unacceptable [64]. Anecdotal experiences with older patients with VPD who have not have surgery due to limited resources in developing countries show that VPD does not resolve spontaneously. Clinical observations indicate that even minor amounts of VPD do not generally correct themselves and tend to increase with age [65].

Patients underwent surgery between the ages of 2.4 and 31 years. One may postulate that those undergoing surgery at an older age may be disadvantaged since compensations are more ingrained and their brains have less plasticity to relearn speaking techniques. Yet, when tested, age was not found to predict speech outcome [57] nor the need for further surgery [47].

All children receive speech and language therapy. When this insufficiently corrects VPD due to anatomic deficits, the velopharyngeal gap can be decreased in size by obturation with a prosthesis, inserting autologous or synthetic materials, or surgically. The clinical and radiological characteristics of the patient and the velopharyngeal function guide the clinician's treatment choice. Prosthetics are bothersome and less effective than surgery, as proven in a randomized controlled trial among syndromic and nonsyndromic children with moderate to severe VPD [66] or with a hypodynamic pharynx [67].

In many studies, the indication for surgery was not specified beyond “VPD.” When the degree of preoperative VPD was reported (n = 13 studies), the lack of a universal scale hampered comparison between studies. However, in three studies the outcomes after different procedures were reported (n = 3 studies)[Hens and Vander Poorten, unpublished data] [24], [48], allowing comparison of baseline VPD between patients that underwent different procedures. In the study by Rottgers et al. [48], patients who primarily underwent a Furlow procedure had an average Pittsburgh Weighted Speech Score of 18.4, while patients who primarily received a PF the average score was 26.8 [48]. In the studies by Hens and Vander Poorten[unpublished data] and Mehendale et al. [24] no group averages were reported, but each patient was rated on a 5 point scale, making it more difficult to summarize the data. In the study by Hens and Vander Poorten[unpublished data], 50% of the patients who underwent a Hynes procedure (n = 2) had severely hypernasal speech, while 65% of the patients who underwent a Honig procedure (n = 17) had severely hypernasal speech. In the study by Mehendale et al. [24], there was one patient with severely hypernasal speech in each group. One patient who underwent both an IVP and a Hynes procedure was not hypernasal and did not have any nasal emission or turbulence preoperatively but only had mild VPD on nasendoscopy. These baseline differences likely affect outcome: a greater degree of preoperative nasalance is prognostic for an increased need for further surgery [47].

### Imaging

At some centers, preoperative imaging is assessed with the assumption that the velopharyngeal closure pattern should dictate the procedure choice [18] or the amount of velopharyngeal movement should affect the operative technique. However, both the assessment of the imaging and the extrapolation to a specific surgical procedure are imperfect. Using standardized assessment of nasendoscopic views of velopharyngeal movement [68], interrater reliability was only 0.4 for semi-quantitative judgment of velar and lateral wall motion, and even lower for characteristics that were measured qualitatively [69]. Similarly, interrater agreement was <0.5 among routine assessors of videofluoroscopy [70], but >0.8 in another center [71], [72].

Furthermore, both the amount and pattern of velopharyngeal motion [17], [39], [70], [71], [73], [74], [75] and the dimensions of a PF [76] change after surgery, compromising the logic of tailoring procedures and/or techniques based on preoperative findings. Among syndromic and nonsyndromic children the amount of lateral wall adduction is not correlated with outcome [76]. In this systematic review, patients who had more favorable velopharyngeal movement underwent less obstructive surgeries (Table S2), Compared to their counterparts who had less favorable velopharyngeal movement and underwent more obstructive pharyngoplasties, fewer patients who underwent less obstructive fat injections or palatoplasties attained normal resonance and more patients required further surgery.

### Surgical procedures

Ideally, an operation is based on anatomic and physiologic knowledge and clinical trials to test the hypothesis [31]. In a cadaver study, Huang et al [22] reason that the palatoplasty is the most physiological solution to restore velopharyngeal function when there is a cleft palate with maloriented muscles as it reinstates the sling mechanism of the levator veli palatini muscles. When there is VPD despite the correct positioning of the palatal muscles, a pharyngoplasty is often required. A SP is said to preserve the sphincter function of the superior constrictor while augmenting the thickness of the pharyngeal walls, decreasing the velopharyngeal port size [46]. Creating a PF, conversely, disrupts the pharyngeal sphincter mechanism by dividing the superior constrictor muscle [22]. However, the flap donor site on the posterior pharyngeal wall heals by circular contraction [77], possibly causing the muscle fibers to migrate medially [78].

The results from trials among patients without 22qDS should not be simply be extrapolated to this unique group [48], [53]. Lipson et al [6] lament that a standard repair of an overt or submucous cleft was never adequate to prevent VPD in patients with 22qDS. Having VPD and any syndrome is associated with having a hypodynamic velopharynx [67] and is prognostic for poorer postoperative outcome [39]. Lower primary success rates for all patients with hypodynamic velopharynges, including those with 22qDS, supports the logic of segregating this group (which constitutes up to 25% of the population with VPD) from the larger cleft palate population [67]. In general, the speech outcome after surgery has been reported to be worse in patients with 22qDS than in patients without the syndrome [25], [36], [47], [49], [56], [79], [80], [81], but some patients with 22qDS fare as well as their non-syndromic counterparts [33], [39], [40], [46], [52], [61], [82].

Treating VPD in any patients with hypo- or adynamic velopharynges, including nonsyndromic patients and patients with other syndromes, is a challenge. A study comparing outcomes reported 42% (n = 15/36) failure after primary treatment among patients with a hypodynamic velopharynx and only 13% (n = 16/119) failure among patients with a dynamic velopharynx [67]. Treatment algorithms suggest creating a SP in patients with a hypodynamic pharynx [15], [23]. However, in patients with neurologic VPD, PFs and SPs have similar outcomes [29], [83].

The choice which surgical technique to employ is largely based on the surgeon's preference [67]. Forty-eight percent of surgeons who answered a questionnaire (n = 13/27) create PFs in over half of their patients with 22qDS [51]. This systematic review confirms this predilection for PFs. Some prefer to create a PF [53], stating the outcome is superior because the procedure is simpler and the results are less variable than after a SP [84]. Others prefer a SP above a wide PF because the latter has an increased risk of OSA [18], [23], [24], [47], [67]. Finally, one center recommends a two-staged approach and waiting six months between a palatoplasty and pharyngoplasty to determine whether the need for a pharyngoplasty has been resolved or allow a less obstructive pharyngoplasty to be created [24], [85].

### Surgical techniques

Not all palatoplasties, PFs, or SPs are the same. A palatoplasty can include a Z-plasty [39], [46], [48], [49], [81] or varying degrees of dissection and repositioning of the levator veli palatini muscles [24]. A PF can be cranially [33], [50] or caudally based [60]. A palatoplasty with supraperiosteal retropositioning of the velar sling can be combined with a PF in a (modified) Honig procedure [37], [41]. The PF donor site can be closed [21], [54], [72], [73], [84] or left to heal by secondary intention [21] thereby allowing scar constriction to decrease the pharyngeal width [71]. The width of PFs can be varied by lining [17], [54] or shortening [61] the flap to prevent tubing [86]. Even then, the eventual flap width is unpredictable [61], [72], [76], [87], compromising the logic of tailoring the technique based on velopharyngeal movement. During an SP, the width of the flaps [25], [53], the height of inset [24], [25], [53], [62] and the amount of overlap of the two lateral flaps [47], [80] can be varied.

In this systematic review, despite the differences in technique, Furlows and IVPs were not separated since both are palatoplasties in which no material is added and the levator veli palatini muscles are positioned as physiologically as possible. SPs and Hynes were not separated since in both procedures lateral flaps are created, rotated, and inset on the posterior pharyngeal wall. For both SP and Hynes, the height of inset was tailored to the level of attempted velopharyngeal contact.

### Outcome

Definitions of success differ [25]. Since the indication for a corrective surgery is VPD, the goal should be resolution of VPD while avoiding overcorrection and the need for further surgery [47]. As Furlow Jr. so strongly stated, “there are no points for ‘significant improvement’ … near-miss successes in one institution may not be classified the same in another; they make inter-institutional comparisons of questionable validity” [39]. Certainly this systematic review has questionable validity due to the differences in reporting between centers. We attempted to bypass the different definitions by including only numbers of patients with normalized resonance. Undoubtedly, the definition of normalcy also differs between centers.

None of the interventions in current use is completely successful in correcting VPD. The low rate of normal resonance may be attributed to the short postoperative follow-up after which the full effect of speech therapy has not yet been achieved [57].

The low rate of normal resonance may reflect the purposeful creation of less obstruction to prevent OSA. OSA is a possible serious complication following pharyngoplasty [65] and is associated with pharyngeal hypotonia [26]. Patients with 22qDS with hypotonia who undergo surgical correction of VPD are therefore particularly at risk for developing OSA [77], [88], [89], [90].

Despite surgeons' fears of inducing OSA, we found only 4 cases in these studies. Interestingly, OSA did not occur more frequently among patients receiving PFs (n = 2) than those receiving SPs (n = 2). In one case, the OSA resolved within 3 weeks on nasally applied continuous positive airway pressure [62]. The others had further surgery to increase the velopharyngeal port size. No OSA occurred when a palatoplasty and pharyngoplasty were performed in one stage [46] nor at centers where the two-stage approach is employed [24], [48].

Further surgery may be needed when there is residual VPD [24], [36], [48], [49], [55], [56], [57], [59], [62] or OSA [44], [47], [54]. Whether it is carried out depends on the recommendation of the cleft team and the patients' or their family's desires [25], [54], [62]. The increased need for further surgery among patients who underwent a palatoplasty is affected by the deliberate two-staged approach.

There were no significant differences in speech outcomes or morbidity between the groups that underwent different types of pharyngoplasties. It is unclear whether this reflects the appropriateness of tailoring based on velopharyngeal movement, or whether the procedures have similar efficacy despite differences in velopharyngeal movement.

### Conclusion

Based on outcomes research (level 2c evidence) and poor quality cohort studies (level 4 evidence), a Grade C recommendation [58] can be made to minimize the morbidity of further surgery for patients with 22qDS and VPD by choosing to perform a pharyngoplasty directly. Only performing a palatoplasty resulted in a greater need for further surgery. Higher level evidence is needed to confirm or refute these findings. While a randomized controlled trial seems unfeasible, by conducting prospective cohort studies at multiple centers and uniformly documenting patient characteristics, velopharyngeal movement, and outcome measures, a meta-analysis could be performed with correction for the various factors.

## Supporting Information

Table S1 **Validity assessment. Criteria based on the Cochrane Collaboration's tool for assessing risk of bias** [**32**] **.**
(DOC)

Table S2 **Imaging modalities and assessments.**
(DOC)
