# Supplementary material for: In Search of the Optimal Surgical Treatment for Velopharyngeal Dysfunction in 22q11.2 Deletion Syndrome: A Systematic Review
Source: PLoS One. 2012 Mar 28;7(3):e34332. doi: 10.1371/journal.pone.0034332 (PMC3314640; doi:10.1371/journal.pone.0034332)
Supplement: Table S1 — Validity assessment. Criteria based on the Cochrane Collaboration's tool for assessing risk of bias [32] . (DOC) [file pone.0034332.s001.doc]

**Table S1**: Validity assessment. Criteria based on the Cochrane Collaboration's tool for assessing risk of bias[32].

| **Study** | **Genetic confirmation** | **Included all patients** | **Randomized** | **Follow-up >1 year** | **Loss to follow-up (%)** | **Blinded** | **Resonance** | **Validated speech test** | **Points (Max 7)** | **Design** | **Level of Evidence** |
| --- | --- | --- | --- | --- | --- | --- | --- | --- | --- | --- | --- |
| *Argamaso 1994[61]* | - | no^a^ | no | yes | - | n/a | yes | - | 2 | Outcomes research | 2c |
| *Arneja 2008[21]* | FISH | no^b^ | no | yes | - | n/a | yes | yes | 4 | Outcomes research | 2c |
| *Baylis 2008[45]* | FISH | no^c^ | no | - | - | yes | yes | yes | 4 | Cohort study | 4 |
| *Brandao 2011[52]* | no (6 clinical signs) | no^e^ | no | - | - | no | yes | yes | 2 | Outcomes research | 2c |
| *d'Antonio 2001[49]* | FISH | no^b^ | no | - | - | - | - | - | 1 | Outcomes research | 2c |
| *Goorhuis 2003[60]* | FISH | yes | - | - | - | n/a | yes | - | 3 | Outcomes research | 2c |
| *Hens and Vander Poorten, unpublished data* | yes | yes | no | yes | 10/29 (34) | no | yes | no | 4 | Outcomes research | 2c |
| *Leuchter 2009[55]* | - | no^b^ | no | no | - | n/a | no | no | 1 | Outcomes research | 2c |
| *Lipson 1991[6]* | yes | yes | no | no | 8/32 (25) | n/a | yes | - | 3 | Cohort study | 4 |
| *Losken 2006[47]* | - | yes | no | - | - | n/a | no | - | 1 | Outcomes research | 2c |
| *MacKenzie 1987[59]* | - | no^d^ | no | - | - | n/a | yes | - | 1 | Cohort study | 4 |
| *Mehendale 2004[24]* | FISH | no^e^ | no | no | 5/47 (11) | yes | yes | yes | 5 | Outcomes research | 2c |
| *Milczuk 2007[46]* | FISH | no^e^ | no | no | 3/14 (21) | yes | no | no | 2 | Outcomes research | 2c |
| *Nicolas 2011[56]* | if available | no^b^ | no | yes | 0/6 (0) | n/a | no | no | 3 | Outcomes research | 2c |
| *Perkins 2005[39]* | no | no^f^ | no | no | - | n/a | no | - | 0 | Outcomes research | 2c |
| *Rottgers 2011[48]* | FISH | yes | no | no | - | no | no | yes | 3 | Outcomes research | 2c |
| *Rouillon 2009[33]* | FISH | no^g^ | no | yes | - | n/a | no | no | 2 | Outcomes research | 2c |
| *Sie 1998[25]* | - | no^b^ | no | no | 6/30 (20) | no | yes | - | 1 | Outcomes research | 2c |
| *Spruijt 2011[57]* | FISH | no^b^ | no | yes | 10/54 (19) | n/a | yes | no | 4 | Outcomes research | 2c |
| *Swanson 2011[54]* | FISH | yes | no | no | 7/40 (18) | n/a | yes | yes | 5 | Outcomes research | 2c |
| *Tatum 2002[35]* | FISH | no^d^ | no | no | 0/20 (0) | n/a | yes | - | 3 | Outcomes research | 2c |
| *Wang 2009[50]* | - | yes | no | - | - | n/a | - | yes | 2 | Outcomes research | 2c |
| *Widdershoven 2008[36]* | FISH | yes | no | - | - | n/a | yes | no | 3 | Outcomes research | 2c |
| *Widdershoven*  *in press[44]* | FISH/MLPA | yes | no | yes | - | no | yes | yes | 5 | Outcomes research | 2c |
| *Witt 1998[51]* | FISH | no^d^ | no | - | - | no | - | - | 1 | Outcomes research | 2c |
| *Witt 1999[62]* | FISH | no^b^ | no | yes | - | n/a | yes | - | 3 | Outcomes research | 2c |
| *Ysuzna 2009[53]* | FISH | no^h^ | no | no | - | no | yes | yes | 3 | Outcomes research | 2c |

-: not reported; a: only those with asymmetric VPD, b: only those undergoing this technique; c: , only those responding to a questionnaire; d: only those with abnormal carotid arteries; e: only those without previous palate surgery; f: only those with sagittally orientated m. levator palatini; g: only those without a palatal anomaly; h: only those without severe neurological disorders or abnormal hearing; n/a: not applicable.
