# Supplementary material for: In Search of the Optimal Surgical Treatment for Velopharyngeal Dysfunction in 22q11.2 Deletion Syndrome: A Systematic Review
Source: PLoS One. 2012 Mar 28;7(3):e34332. doi: 10.1371/journal.pone.0034332 (PMC3314640; doi:10.1371/journal.pone.0034332)
Supplement: Table S2 — Imaging modalities and assessments. (DOC) [file pone.0034332.s002.doc]

**Table S2: Imaging modalities and assessments.**

| **First author, publication year** | **Procedure** | **Imaging modality** | **Pharyngeal lateral wall movement** | **Velar movement** | **Gap size** | **Closure pattern** | **Tailored** |
| --- | --- | --- | --- | --- | --- | --- | --- |
| *Leuchter 2009[55]* | fat injection | N | - | good | minimal | - | yes |
| *Nicolas 2011[56]* | fat injection | - | - | - | - | - | yes |
| *Milczuk 2007[46]* | Furlow | N | % | % | <50% | yes | yes |
| *d'Antonio 2001[49]* | Furlow | N + X | - | good | minimal | - | yes |
| *Rottgers 2011[48]* | Furlow | N + X | - | good | - | - | yes |
| *Perkins 2005[39]* | Furlow | N | % | % | 3 ps | no | yes |
| *Mehendale 2004[24]* | IVP | X | - | V* | yes | - | yes |
| *Brandao 2011[52]* | IVP | N | - | - | 6 ps | - | - |
| *MacKenzie 1987[59]* | PF | N + X | - | - | - | - | yes |
| *Argamaso 1994[61]* | PF | N + X | yes | - | - | - | yes |
| *Baylis 2008[45]* | PF | - | - | - | - | - | - |
| *Rottgers 2011[48]* | PF | N + X | - | poor | - | - | yes |
| *Witt 1998[51]* | PF | N + X | satisfactory | poor | narrow to moderate | yes | yes |
| *Brandao 2011[52]* | PF | N | - | - | 6 ps | - | - |
| *Arneja 2008[21]* | PF | N + X | yes | yes | yes | - | - |
| *Rouillon 2009[33]* | PF | N | - | - | - | - | - |
| *Goorhuis 2003[60]* | PF | - | - | - | - | - | - |
| *Tatum 2002[35]* | PF | N + X | % | % | - | - | yes |
| *Ysunza 2009[53]* | PF | N + X | % | % | % | yes | yes |
| *Swanson 2011[54]* | PF | X | % | - | 3 ps | yes | yes |
| *Wang 2009[50]* | PF | X | yes | - | yes | no | yes |
| *Lipson 1991[6]* | (likely PF) | X | - | yes | - | - | - |
| *Widdershoven in press[44]* | PF (33), SP (7) | N + X | 3 ps | 3 ps | yes | yes | yes |
| *Rottgers 2011[48]* | PF + Furlow | N + X | - | good | - | - | yes |
| *Hens and Vander Poorten, unpublished data* | Honig | N + X | yes | yes | - | - | no |
| *Widdershoven 2008[36]* | Honig | N | 4 ps | 4 ps | 4 ps | - | no |
| *Spruijt 2011[57]* | Honig | N | 3 ps | 3 ps | - | - | no |
| *Baylis 2008[45]* | SP | - | - | - | - | - | - |
| *Milczuk 2007[46]* | SP | N | % | % | large | yes | yes |
| *Sie 1998[25]* | SP | N + X | 6 ps | 6 ps | 3 ps | yes | yes |
| *Witt 1998[51]* | SP | N + X | poor | poor | large | yes | yes |
| *Ysunza 2009[53]* | SP | N + X | % | % | % | yes | yes |
| *Witt 1999[62]* | SP | N + X | poor | active | large | yes | yes |
| *Losken 2006[47]* | SP | N + X | yes | yes | yes | yes | yes |
| *Milczuk 2007[46]* | SP + Furlow | N | % | % | >50% | yes | yes |
| *Hens and Vander Poorten, unpublished data* | Hynes | N + X | yes | yes | - | - | - |
| *Sie 1998[25]* | Hynes | N + X | 6 ps | 6 ps | 3 ps | yes | yes |
| *Mehendale 2004[24]* | Hynes | N + X | - | V* | large | - | yes |
| *Mehendale 2004[24]* | Hynes + IVP | N + X | - | V* | large | - | yes |

- : not reported; %: measured as a percentage value; I: improvement; IVP: intravelar veloplasty; N: nasendoscopy; PF: pharyngeal flap; likely PF: pharyngoplasty not otherwise specified; ps: point scale; SP: sphincter pharyngoplasty; V*: closure ratio, extended length, velocity of closure, lift; X: X-ray cephalograms or (video)fluoroscopy.
